# Pregabalin treatment in a 30-year-old patient with Bainbridge-Ropers syndrome: a case-report

**DOI:** 10.3389/fpsyt.2024.1502773

**Published:** 2024-12-04

**Authors:** Marie Geiser, Jean-Marc Good, Vincent Guinchat

**Affiliations:** ^1^ Département de Psychiatrie, Centre Hospitalier Universitaire Vaudois (CHUV), Lausanne, Switzerland; ^2^ Ecole de médecine, Université de Lausanne, Lausanne, Vaud, Switzerland

**Keywords:** Bainbridge-Ropers syndrome, ASXL3, pregabalin (PGB), autism, anxiety, epilepsy

## Abstract

Mr. X is a Swiss patient with Bainbridge-Ropers syndrome clinically and genetically diagnosed at the age of 28. He is also known to have severe intellectual disability, autism spectrum disorder and epilepsy since the age of 18. At the age of 30, he was admitted for the first time to a psychiatric crisis unit dedicated to mental disabilities for challenging behavior such as self-aggression (forceful vomiting, scratching himself, pulling out his toe and fingernails or banging his head against the wall), agitation, screaming, dropping to the ground, damaging electronic items, or even displaying hetero-aggressive gestures (trying to bite or pull hair, scratching, kicking, or punching) associated with a drop in mood, withdrawal from usual activities, a drop in social interaction and a tendency to doze off during the day. The introduction of Pregabalin leads to rapid stabilization of the clinical state, almost complete improvement in challenging behavior and gradual withdrawal of other treatments (class 2 analgesics, neuroleptics, antidepressants, and benzodiazepines). At the neurological check-up 9 months after discharge from hospital, clinical stability was confirmed by the surrounding team and the medical observation, with almost complete disappearance of auto-aggressive gestures.

## 1 Introduction

Bainbridge-Ropers syndrome (BRS) is an autosomal dominant genetic syndrome characterized by loss-of-function mutations in the Additional sex combs-like 3 (ASXL3) gene (18q12.1). It is a genetic syndrome recently discovered (2013) in the context of the genetic analysis of patients presenting with clinical features compatible with a diagnosis of Boring-Opitz syndrome. BRS is a rare syndrome with a prevalence of less than 1/1,000,000. No specific management or targeted treatment is currently known. The literature mostly focus on genetic diagnosis and clinical descriptions, which are based on non-specific descriptive signs (1, 2). They include developmental delay or moderate-to-severe intellectual disability with delayed or absent language skills, autism spectrum disorder, non-specific facial dysmorphia, hypotonia which may evolve into spasticity with postural patterns, notably the flexion of elbows, wrists, and fingers. Other signs include growth disorders, feeding difficulties in early childhood, strabismus, behavioral disorders, epilepsy, sleep disorders with increased risk of sleep apnea linked to hypotonia, and dental anomalies (3–5).

The mutation causing this syndrome is a truncated variant affecting the gene responsible for the production of the ASXL3 protein, resulting in a reduction of ASXL3 activity (1). The function of this protein in humans is not yet well defined, but it is strongly involved in the mammalian brain development, especially in the cell fate specification during early neural development (6–8).

Consensual clinical management guidelines for ASXL3-related disorder have not yet been published. Treatment is usually symptomatic, with multidisciplinary management based on the most problematic symptoms (9).

## 2 Case presentation

Mr. X is a 30-year-old male patient, Swiss, Caucasian, from a non-consanguineous union. He is the second child of two siblings. The pregnancy and delivery are said to be unremarkable, with the patient’s development said to be linear during the first three months of life, feeding difficulties with vomiting after bottle-feeding and no notable somatic disorders to be found. The patient subsequently showed a progressive form of overall delay in development associated with autism spectrum disorder, facial dysmorphia, hypotonia, strabismus and behavioral disorders. He therefore joined the children’s sector of a social institution a few days a week and then for school days from the age of five onwards. Medically the patient is known for epilepsy since the age of 18 with notion of generalized tonic-clonic seizures and myoclonic seizures. At the age of 28, Mr. X was hospitalized in a neurological unit for status epilepticus resulting in an increase in his antiepileptic medication.

In the months following this hospitalization, the patient beneficiated from a genetic consultation given his clinical features which associated the various specific signs listed above. Following the genetic analysis, the diagnosis of Bainbridge-Ropers syndrome was made.

Regarding behavioral disorders, he has occasionally presented, since childhood, challenging behaviors, occurring periodically, without any obvious triggers and with spontaneous resolution. During these episodes, he may present with psychomotor agitation, screaming, dropping to the ground, damaging electronic items, or even displaying hetero-aggressive gestures (trying to bite or pull hair, scratching, kicking, or punching), or self-harming (forceful vomiting, scratching himself or banging his head against the wall).

Since the age of 28, he would exhibit such behavioral problem at an increasing rate of several times per day. He would also exhibit new problematic behaviors, such as pulling out his toe and fingernails. The patients’ relatives describe a decline in his mood, along with him becoming withdrawn, a refusal to participate in activities, a drop in social interaction and a tendency to doze off during the day. Eating habits fluctuated, with alternating periods of food refusal and minor hyperphagia. His relatives also described episodes of hyperventilation, occurring since childhood but having increased in frequency and intensity over the recent months, particularly during periods of transition. Finally, the patient presented with a long-standing sleeping disorder, waking frequently during the night, and vocalizing in his sleep.

In view of this behavioral deterioration Fluvoxamine 150 mg/d and Risperidone 1 mg/d were introduced, with no clear improvement in symptomatology, leading to the discontinuation of Risperidone replaced by Olanzapine 10 mg/d without any clinical improvement. These are the treatments recommended to treat symptoms of irritability in patients with autism. The introduction of a treatment of proton pump inhibitor (IPP) 40 mg/j with the hypothesis of gastric reflux pain, which might explain certain challenging behaviors (eating disorders, torso laceration, etc.) led to a slight improvement in hetero-aggressive behaviors, but only transiently. Given the significant clinical deterioration over the previous month, with the onset of self-induced injuries, a tramadol-based treatment 200 mg/d was introduced with the hypothesis of dolor, in parallel to Fluvoxamine and Olanzapine, without any clinical improvement.

In view of the severity and frequency of these challenging behaviors over the previous months, he was admitted to a psychiatric crisis unit dedicated to mental disabilities. This was the patient’s first psychiatric hospitalization.

Usual treatment:

Sodium Valproat tablet: 500 mg morning, 500 mg midday, 1000 mg evening

Lacosamide: 100 mg morning, 100 mg evening

Olanzapine: 10 mg/d

Fluvoxamine: 150 mg/d

Tramadol retard: 100 mg morning, 100 mg evening

Esomeprazole: 40 mg/d

Daily: Alprazolam 0.25 mg treatment for behavioral crises in reserve maximum twice a day

## 3 Hospital management

On his arrival, Mr. X presented several episodes of hyperventilation per day (with varying contexts: when a caregiver would address him, at the start of meals, during transitions or sometimes without any context), extremity tremors, provoked vomiting, repeated questions (“who is that?”, “what is that?”, “tomorrow”), regular episodes of screaming and refusal of care. These episodes pointed to a manifestation of anxiety. At the same time, the patient presented with self-aggressive behaviors such as lacerations of the torso, pulling of nails, toes, and fingers, or thermal stimulation by putting his hands under very hot water for several minutes at a time. He also presented with phases of agitation during which he would urinate on electrical outlets, spills water on the floor or on electronic equipment, try to set off alarms, or even display hetero-aggressive gestures (grabbing caregivers’ hair, hitting them with feet or hands). On clinical examination, vital parameters were within normal limits (weight: 54 kg; blood pressure: 108/65 mmHg; heart rate: 71 bpm; oxygen saturation: 100%; temperature: 36.2°C). Severe intellectual disability with language delay, autism, facial dysmorphia with prominent forehead, arched eyebrows and serrated teeth, strabismus, generalized hypotonia associated with skeletal malformations: Marfanoid habitus, pectus excavatum and arachnodactyly were noted. The remainder of the clinical examination revealed self-induced nail-biting lesions on left index finger and all toes without any signs of localized infection. No cardiac, pulmonary or abdominal abnormalities were noted. Neurologically, on two occasions the patient presented with clinical manifestations strongly suggestive of epileptic seizures, despite taking his usual treatment as prescribed. Neurological observation and electroencephalogram evaluation subsequently returned to normal, suggesting an uncertain origin for these seizures. Treatment with brivaracetam 100 mg/d was added, with the aim of improving control of epilepsy. In view of the marked deterioration in the patient’s behavior under Brivaracetam (psychomotor agitation with increased insomnia, major aggressiveness towards others and himself, endangerment by repeated urination onto electrical outlets, etc.) and the absence of any clear argument from the neurology specialists for potential benefits, Brivaracetam was quickly withdrawn.

Regarding gastrointestinal symptoms, the patient presented several episodes of induced vomiting at the beginning of his stay, as well as episodes of hyperventilation before meals, possibly compatible with digestive discomfort. An abdominal scanner and oeso-gastro-duodenal transit were performed in addition to an extensive laboratory work-up. Imagery examens were in the norm. Biological tests showed no obvious abnormalities.

Faced with these clinical features, and in particular the atypical self-aggressive gestures directed at his fingernail, we elaborated on the hypothesis of pain linked to an inflammatory disease of the extremities, as part of the Bainbridge-Ropers syndrome. Treatment with non-steroidal anti-inflammatory (AINS) ex officio (Ibuprofen 200 mg three times a day) was started with improvement in self-harm behaviors during three days, but with no noticeable effect on other symptoms of agitation.

To reduce the risk of iatrogenicity, Tramadol and Fluvoxamine treatments were reduced and then stopped, in view of their lack of effectiveness on behavior when they were first introduced. The Olanzapine treatment was also reduced, then replaced by a low-dose Risperidone treatment (0.5 mg/d).

As the patient had already benefited from first-line treatments for irritability in autistic patients, antidepressants for the hypothesis of an anxiety disorder diagnosis, as well as from the implementation of a conventional psycho-educational program in a patient presenting with behavioral disorders with no observed clinical effect, the differential diagnosis of the following 3 comorbidities was considered in view of the patient’s clinical presentation: generalized anxiety disorder (episodes of hyperventilation, extremity tremors, provoked vomiting, repeated questions, regular episodes of screaming and refusal of care) (10), poorly controlled epilepsy and chronic neuropathic pain. However, since epileptic manifestations present atypically (maintenance of consciousness, tonic movement of the trunk without clonic movements) and EEGs show no visible abnormalities, these manifestations may also be part of behavioral symptoms related to primary or secondary anxiety. The patient’s behaviors suggestive of chronic neuropathic pain (nail biting, hand burning, self-mutilation) led us to diagnose an anxiety disorder secondary to chronic neuropathic pain as the main diagnosis.

In view of this main diagnosis and these 3 differential diagnoses, we chose to introduce a molecule with a pleiotropic effect on anxiety, epilepsy and chronic neuropathic pain. We introduced a treatment with Pregabalin at an initial dose of 75 mg Q.d, doubling the dose every 5 days up to 600 mg Q.d. The introduction of Pregabalin was accompanied by a marked and rapid improvement in behavior, with significant regression in the frequency of self-aggressive gestures, episodes of self-induced vomiting and hyperventilation as well as the disappearance of episodes of hetero-aggression, destruction or urination on objects, especially electrical ones. Mr. X’s behavior became increasingly appropriate, he was gradually able to take part in activities by himself or in a group. The Risperidone medication was stopped, without resurgence of problematic behaviors. The patient underwent a rheumatology consultation, which confirmed the presence of the hyperlaxity syndrome and, in the context of this syndrome, probable peripheral neuropathies. By the end of the hospital stay, the patient’s residual symptoms were sleeping disturbances, improved by a treatment with of small doses of mirtazapine (15 mg/d). Somatically, following the introduction of pregabalin, the patient no longer presented with epileptic-like manifestations and episodes of vomiting regressed significantly. A few days before the patient was discharged from hospital, a new challenging behavior emerged: the patient presented episodes of fixation on female caregivers, seeking inappropriate physical contact (hair, face, private parts) and obsessively seeking the caregiver’s exclusive attention. For example, if the caregiver on whom the patient has an obsession was leaving the room through a door, the patient remained behind this door for several hours at a time, banging on the door with his hands and feet. Prompt psycho-educational care, including verbal re-framing and behavioral extinction, quickly enabled to reduce the frequency and intensity of these fixations, without eliminating them altogether. This emergence at the end of hospitalization of fixations on the female caregivers could be part of a search for attention linked to an anxiety that is sometimes heightened in certain circumstances.

The patient was overall calm when discharged from the hospital, cooperating in his care, with no episodes of hyperventilation as presented in the beginning of his stay. He had a good thymia presenting with smiles and attempts to bond with caregivers. Mr. X continued to refuse certain activities, although he took part in a variety of individual and group activities (listening to music, swimming, sharing meals with other patients, etc.). He spent long periods of calm, observing his surroundings. We should mention the persistence of a few self-harm behaviors towards his toenails (right and left), but only on occasion over the last two weeks of his hospital stay and mainly in a context of frustration with deviant communication. The patient showed no hetero-aggressive gestures. He expressed himself through a few words (with a restricted lexical field and repeated verbal requests), and through pictograms. He no longer presented appetite or sleep disorders.

Treatment at discharge:

Pregabalin: 200 mg morning, 200 mg midday, 200 mg evening

Sodium Valproat: 500 mg morning, 500 mg midday, 1000 mg evening

Lacosamide: 100 mg morning, 100 mg evening

Mirtazapine: 15 mg evening

Nexium: 40 mg/d

To summarize, treatment with pregabalin, in this 30-year-old patient with Bainbridge-Ropers syndrome, permitted a clear and rapid improvement in behavior with significant regression in the frequency of self-harm gestures, hyperventilation, and disappearance of episodes of hetero-aggression, destruction or urination on objects. It also enabled complete withdrawal from neuroleptics and tramadol, cessation of antidepressant treatment with Fluvoxamine (although a small dose of Mirtazapine for sleep persisted), and cessation of daily benzodiazepine reserve medication. Somatically, it allowed the disappearance of clinical manifestations strongly suggestive of epileptic seizures, and the disappearance of episodes of induced vomiting.

**Figure 1:**
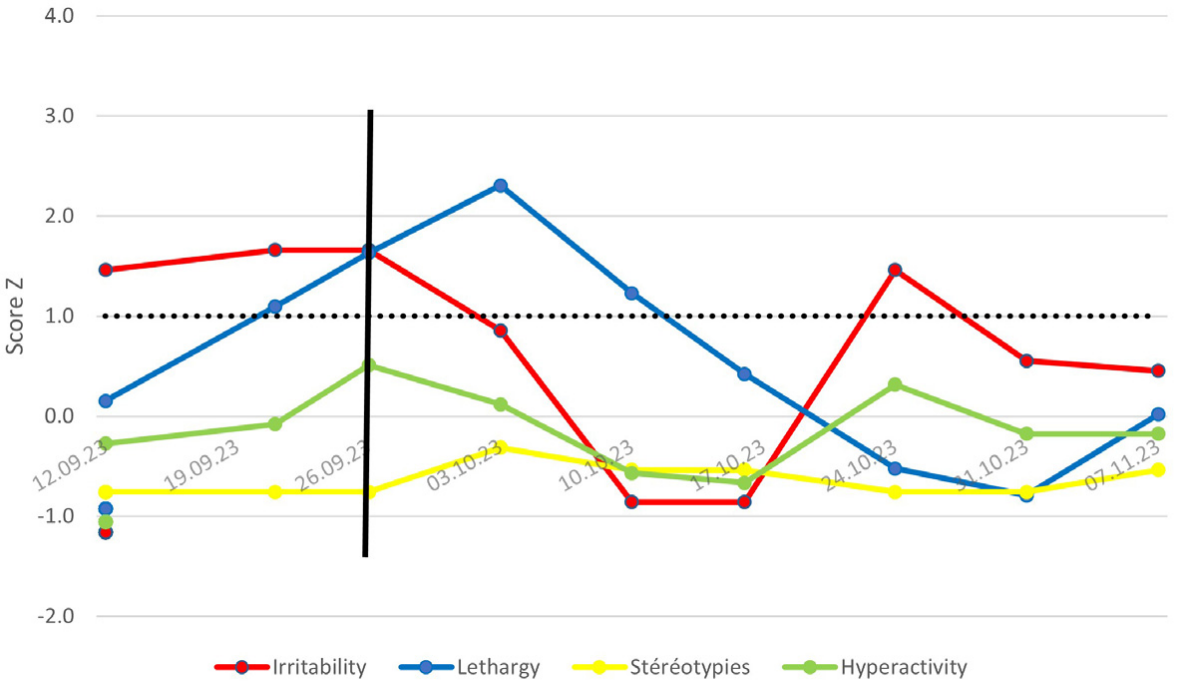
Evolution of ABC scores or Mr. X – Comparison with a population with SID.

**Figure 1** shows the week-by-week evolution of the patient’s ABC score (Antecedents, Behavior, Consequences) during hospitalization. This score is assessed by the care team in direct contact with the patient, based on a list of around sixty questions evaluating his or her behavior over the past week. Only values above the Z-score baseline of 1.0 are significant in comparison with the population with severe intellectual disability (SID).

Following discharge from hospital, the patient returns to live in his usual social institution. On his return, educational support and occupational activities were resumed. Overall, the patient remained clinically stable. He exhibited episodically challenging behaviors that he had experienced in the past, but which did not require any change in medication or adaptation of his daily routine. These episodes appeared to be mainly attention-seeking and did not endanger the patient. At the neurological check-up 9 months after discharge from hospital, clinical stability was confirmed by the surrounding team, with almost complete disappearance of auto-aggressive gestures. Mr. X was described as quiet and cooperative, with an episode of hyperventilation observed but usual in the context of a medical examination. A weight gain of around 10 kilograms and a tendency to slow down language with bradykinesia were noted.

## 4 Discussion

Due to the rarity of the syndrome, there is currently insufficient knowledge to propose recommendations for the specific management of patients with BRS, apart from proposing a multidisciplinary approach focused on the difficulties presented by the patient. This situation can lead to over-medicalization of patients, with a high risk of iatrogenicity associated with the multiplication of drug treatments. The clinical situation of Mr. X and the treatment with pregabalin, which allowed the almost complete cessation of all other psychiatric treatments, seem to us to be important to highlight in order to propose a therapeutic approach to this rare genetic syndrome.

Nevertheless, the complexity of the clinical presentation, the severe intellectual deficits and the patient’s almost non-verbal behavior make it difficult to establish a clear diagnosis of his acute behavioral regression. The first hypothesis could be a generalized anxiety disorder with several signs suggestive of anxiety (hyperventilation, trembling, agitation, etc.). A second hypothesis could be peripheral neuropathic pain in the context of a hyperlaxity syndrome. And a third hypothesis could be a poorly stabilized epilepsy with residual epileptic seizures that may have gone unnoticed by those around the patient, but which may be experienced as unpleasant by the patient, note that hyperventilation episodes can trigger epileptic seizures, so the anxiety and epilepsy hypotheses are intertwined. Pregabalin’s high affinity for the α2δ-1 and α2δ-2 subunits of high-voltage calcium channels enables it to reduce neurotransmission, with an indirect effect on the GABAergic effect. It has a recognized antiepileptic effect, as well as reducing chronic neuropathic pain. This same central effect explains pregabalin’s effect on generalized anxiety (11–13). These three mechanisms all point to the central action of pregabalin. In all three of the above diagnostic hypothesis concerning the patient, pregabalin is an appropriate treatment for each of these conditions.

Fact that pregabalin had such a marked and rapid effect on this patient’s challenging behaviors raises the question of a specific effect on patients with BRS in relation to the genetic mutation. There is no clear link to found in the literature between the effect of pregabalin on the central nervous system and the effect of the ASLX3 protein (11–13), but the pregabalin’s mechanism of action being very wide, notably at the neuronal level, the question of whether the benefits of pregabalin treatment in Bainbridge-Ropers syndrome is confirmed in other cases deserves to be explored. One of the links that could be found is that of ubiquitination failure: this is a pleiotropic phenomenon also responsible for neuropathic pain on which pregabalin acts directly, a phenomenon also linked to the ASXL3 mutation responsible for BRS (8, 12, 14, 15). This lack of knowledge concerning the direct link between BRS and the effect of pregabalin represents the main limitation of this case report. However, this is the first documented case in the literature of the net efficacy of pregabalin treatment in a patient with autism and intellectual deficiency. Whether pregabalin is effective on patients with behavioral disorders, autism or BRS, new research is needed to determine the effect of this treatment on these pathologies.

## 5 Conclusion

The introduction of pregabalin in this particular case of a patient with SBR and autism has led to a clear and lasting clinical stabilization. Whether this is a central effect or a pleiotropic effect on a set of syndrome-related manifestations, the question remains unanswered. However, the value of this treatment remains to be assessed for patients with BRS and autism, with the aim of providing specific, individualized care to meet the needs of these patients.

## Data Availability

The original contributions presented in the study are included in the article/supplementary material. Further inquiries can be directed to the corresponding author.

## References

[1] Bainbridge MN Hu H Muzny DM Musante L Lupski JR Graham BH . *De novo* truncating mutations in ASXL3 are associated with a novel clinical phenotype with similarities to Bohring-Opitz syndrome. Genome Med. (2013) 5:11. doi: 10.1186/gm415 23383720 PMC3707024

[2] Orphanet: Bainbridge-Ropers syndrome (2024). Available online at: https://www.orpha.net/en/disease/detail/352577 (Accessed March 24, 2024).

[3] Ikekwere JC Osuagwu FC LePlatte D Ghaziuddin M . Comorbid psychiatric aspects of bainbridge-ropers syndrome. Prim Care Companion CNS Disord. (2021) 23:34115. doi: 10.4088/PCC.20m02783 34086428

[4] Kuechler A Czeschik JC Graf E Grasshoff U Hüffmeier U Busa T . Bainbridge–Ropers syndrome caused by loss-of-function variants in ASXL3: a recognizable condition. Eur J Hum Genet. (2017) 25:183–91. doi: 10.1038/ejhg.2016.165 PMC5255962 27901041

[5] Schirwani S Woods E Koolen DA Ockeloen CW Lynch SA Kavanagh K . Familial Bainbridge-Ropers syndrome: Report of familial ASXL3 inheritance and a milder phenotype. Am J Med Genet A. (2023) 191:29–36. doi: 10.1002/ajmg.a.v191.1 36177608 PMC10087684

[6] Lichtig H Artamonov A Polevoy H Reid CD Bielas SL Frank D . Modeling bainbridge-ropers syndrome in xenopus laevis embryos. Front Physiol. (2020) 11:75. doi: 10.3389/fphys.2020.00075 32132929 PMC7040374

[7] Micol JB Abdel-Wahab O . The role of additional sex combs-like proteins in cancer. Cold Spring Harb Perspect Med. (2016) 6:a026526. doi: 10.1101/cshperspect.a026526 27527698 PMC5046687

[8] Srivastava A McGrath B Bielas SL . Histone H2A monoubiquitination in neurodevelopmental disorders. Trends Genet. (2017) 33:566–78. doi: 10.1016/j.tig.2017.06.002 PMC5562288 28669576

[9] Balasubramanian M Schirwani S . ASXL3-related disorder. In: Adam MP Feldman J Mirzaa GM Pagon RA Wallace SE Bean LJ , editors. GeneReviews^®^ . University of Washington, Seattle, Seattle (WA (1993). Available at: http://www.ncbi.nlm.nih.gov/books/NBK563693/. 33151654

[10] www.rcpsych.ac.uk . Anxiety and generalised anxiety disorder (GAD) (2024). Available online at: https://www.rcpsych.ac.uk/mental-health/mental-illnesses-and-mental-health-problems/anxiety-and-generalised-anxiety-disorder-(gad) (Accessed October 30, 2024).

[11] Greenblatt HK Greenblatt DJ . Gabapentin and pregabalin for the treatment of anxiety disorders. Clin Pharmacol Drug Dev. (2018) 7:228–32. doi: 10.1002/cpdd.v7.3 29579375

[12] Alles SRA Cain SM Snutch TP . Pregabalin as a pain therapeutic: beyond calcium channels. Front Cell Neurosci. (2020) 14:83. doi: 10.3389/fncel.2020.00083 32351366 PMC7174704

[13] Sills GJ . The mechanisms of action of gabapentin and pregabalin. Curr Opin Pharmacol. (2006) 6:108–13. doi: 10.1016/j.coph.2005.11.003 16376147

[14] Cheng J Deng Y Zhou J . Role of the ubiquitin system in chronic pain. Front Mol Neurosci. (2021) 14:674914. doi: 10.3389/fnmol.2021.674914 34122010 PMC8194701

[15] Marangoudakis S Andrade A Helton TD Denome S Castiglioni AJ Lipscombe D . Differential ubiquitination and proteasome regulation of caV2.2 N-type channel splice isoforms. J Neurosci. (2012) 32:10365–9. doi: 10.1523/JNEUROSCI.0851-11.2012 PMC3428229 22836269

